# GelMA hydrogel enhances diabetic wound healing by delivering shock wave-preconditioned BMSC-derived small extracellular vesicles

**DOI:** 10.3389/fimmu.2026.1971302

**Published:** 2026-10-28

**Authors:** Rongqi Zhou, Xiaoping Xie, Xudong Li, Songqi Bi, Zhixing Yan, Xiaoyang Zhou, Fanxu Wang, Tiecheng Yu

**Affiliations:** 1 Department of Orthopedics, Orthopedics Center, The First Hospital of Jilin University, Jilin University, Changchun, Jilin, China; 2 Department of Sports Medicine, Hubei Sports Medicine Center, Wuhan Fourth Hospital, Wuhan, China

**Keywords:** diabetic wound healing, macrophage polarization, NF-κB signaling pathway, shock wave preconditioning, small extracellular vesicles

## Abstract

**Introduction:**

Diabetic foot ulcers (DFUs) are chronic, hard-to-heal wounds characterized by impaired angiogenesis, persistent inflammation, and defective extracellular matrix remodeling. Bone marrow mesenchymal stem cell-derived small extracellular vesicles (BMSC-sEVs) hold promise for cell-free therapy; however, their therapeutic efficacy is limited by insufficient reparative bioactivity and rapid local clearance. This study aimed to enhance the intrinsic therapeutic potential of BMSC-sEVs through shock wave preconditioning and improve their local retention and sustained delivery using gelatin methacryloyl (GelMA) hydrogel.

**Methods:**

BMSCs were preconditioned with shock waves, and the resulting small extracellular vesicles (SW-sEVs) were isolated and characterized. Their angiogenic and immunomodulatory effects were evaluated using human umbilical vein endothelial cells (HUVECs) and RAW264.7 macrophages. Proteomic analysis and Western blotting were performed to investigate potential underlying mechanisms. SW-sEVs were subsequently incorporated into a 15% GelMA hydrogel, and the resulting delivery system was characterized. Its therapeutic efficacy was evaluated in a streptozotocin (STZ)-induced hyperglycemic rat wound model.

**Results:**

Shock wave preconditioning did not alter the typical morphology, particle size distribution, or marker protein expression of BMSC-sEVs. Compared with sEVs derived from untreated BMSCs, SW-sEVs significantly enhanced HUVEC proliferation, migration, and tube formation, accompanied by increased VEGFA and CD31 expression. SW-sEVs also suppressed pro-inflammatory macrophage activation, promoted a reparative macrophage phenotype, and reduced the expression of pro-inflammatory factors. Proteomic analysis revealed alterations in sEV protein cargo, with differentially expressed proteins enriched in inflammation-related pathways, including NF-κB signaling. Western blotting further demonstrated reduced phosphorylation of p65 and IκBα following SW-sEV treatment. The 15% GelMA hydrogel exhibited an interconnected porous structure, favorable cytocompatibility, structural stability, and sustained SW-sEV release. In vivo, GelMA-SW-sEVs significantly accelerated diabetic wound healing, enhanced neovascularization and M2 macrophage polarization, reduced local inflammation, and improved re-epithelialization, collagen deposition, and tissue remodeling.

**Discussion:**

Shock wave preconditioning enhances the angiogenic and immunomodulatory activities of BMSC-sEVs, potentially through modulation of NF-κB-related inflammatory signaling. Combined with sustained local delivery using GelMA hydrogel, SW-sEVs demonstrate promising therapeutic potential as a cell-free strategy for diabetic wound repair.

## Graphical Abstract

**Figure:**
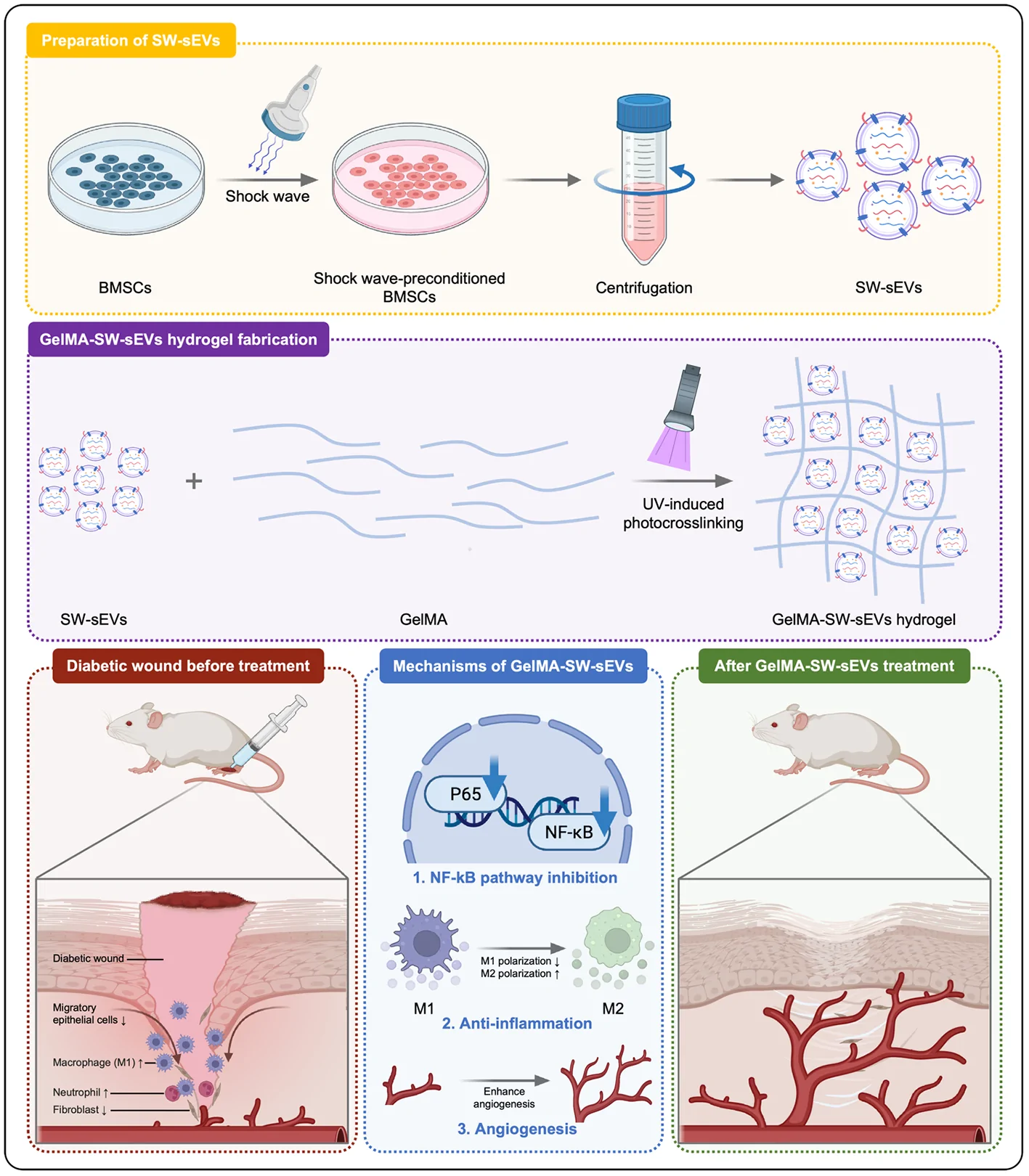
This study developed a methacrylate-modified gelatin (GelMA) hydrogel delivery system loaded with small extracellular vesicles (SW-sEVs) derived from shockwave-pretreated bone marrow mesenchymal stem cells to promote diabetic wound healing. First, bone marrow-derived mesenchymal stem cells were subjected to shock wave pretreatment, and SW-sEVs were isolated; these were then loaded into the GelMA hydrogel, which was cross-linked via UV light to form a GelMA-SW-sEVs composite hydrogel, enabling the sustained local release of SW-sEVs. Mechanistically, SW-sEVs reduce the inflammatory response at the wound site by inhibiting the activation of the NF-κB signaling pathway, reducing p65-related inflammatory signals, suppressing M1-type macrophage polarization, and promoting their conversion to the M2-type reparative phenotype. At the same time, this delivery system enhances angiogenesis, improves local microcirculation at the wound site, and promotes tissue regeneration. *In vivo* results indicate that GelMA-SW-sEVs promote re-epithelialization, angiogenesis, and tissue remodeling, ultimately accelerating the healing of diabetic wounds.

## 1 Introduction

Diabetic foot ulcers (DFUs) are among the most serious chronic complications of diabetes, markedly increasing the risks of infection, lower-limb amputation, and death while severely impairing quality of life (1). DFU development involves multiple pathological processes that remain incompletely understood. Current evidence indicates that hyperglycemia-induced endothelial dysfunction, together with persistent macrophage polarization toward the pro-inflammatory M1 phenotype, drives prolonged inflammation, impaired vascular regeneration, and delayed wound healing (2–4). Clinical management mainly includes glycemic control, debridement, anti-infective therapy, negative-pressure wound therapy, functional dressings, and revascularization (5). Although these interventions improve outcomes to some extent, stable and complete healing remains difficult in patients with chronic inflammation, microcirculatory dysfunction, and impaired tissue regeneration (6). Therefore, therapeutic strategies that simultaneously promote angiogenesis and regulate the immune microenvironment are urgently needed (7).

Extracellular vesicles derived from diverse cellular sources, including platelets, immune cells, endothelial cells, and mesenchymal stem cells (MSCs), have been investigated as mediators or therapeutic candidates in wound repair (8–11). Blood- and platelet-derived EVs are readily accessible and have been shown to promote fibroblast proliferation and migration as well as endothelial angiogenic activity (9), whereas immune cell- and endothelial cell-derived EVs exert source-dependent immunomodulatory and tissue-reparative effects (8, 10). MSC-derived small extracellular vesicles (MSC-sEVs), however, are particularly attractive because of their low immunogenicity, broad paracrine activity, and the ability to expand and precondition parental MSCs ex vivo to modulate vesicle bioactivity (12). MSC-sEVs can regulate macrophage phenotypes, attenuate inflammation, and promote angiogenesis and tissue repair (13). Among MSC sources, BMSCs are widely used in regenerative medicine and are highly responsive to physical and mechanical stimulation, making them particularly suitable for preconditioning-based modulation of sEV production and cargo (14). Nevertheless, the clinical translation of MSC-sEVs remains limited by relatively low production yields, insufficient intrinsic bioactivity, rapid degradation or clearance after administration, and poor retention at the wound site (15). In the present study, shock wave preconditioning was therefore used to enhance the biological activity of BMSC-sEVs, while GelMA hydrogel was employed to prolong their local retention and sustained delivery.

Mechanical stimulation is an important microenvironmental cue that regulates stem cell fate, secretory activity, and paracrine function (16). As a controllable mechanical biostimulus, shock waves (SW) can activate mechanosignaling and reshape the secretory phenotype of BMSCs (17). Low-energy shock waves have been shown to promote vascular endothelial growth factor receptor 2 (VEGFR2) phosphorylation and receptor cycling in endothelial cells, thereby enhancing angiogenesis (18). They can also suppress M1-associated inflammatory genes and interleukin-1β (IL-1β) expression in macrophages while increasing interleukin-10 (IL-10) and selected M2-associated markers, thus shifting the inflammatory response from a persistent pro-inflammatory state toward resolution and tissue repair (19). Based on these effects, SW pretreatment may enhance the immunomodulatory and angiogenic properties of BMSC-derived sEVs by regulating cellular secretory activity (20). However, whether sEVs derived from SW-pretreated BMSCs (SW-sEVs) can effectively ameliorate impaired angiogenesis and persistent inflammation in diabetic wounds remains insufficiently investigated. In addition to limited bioactivity, locally administered sEVs readily diffuse or are rapidly cleared, resulting in short wound-site residence and inadequate effective concentrations (17). Hydrogel-based delivery can protect sEVs, prolong local retention, and enable sustained release (21). Gelatin methacryloyl (GelMA), a widely used photocrosslinkable hydrogel precursor derived from modified gelatin, offers good biocompatibility, degradability, injectability, and structural tunability (22). Its three-dimensional crosslinked network and porous architecture facilitate sEV loading, local retention, and sustained release while maintaining a moist and supportive microenvironment for wound repair (23). In the present study, GelMA was therefore used as an established local delivery matrix to improve the retention and sustained release of functionally enhanced SW-sEVs, thereby complementing the enhanced bioactivity achieved through shock wave preconditioning.

In this study, we investigated whether shock wave preconditioning could enhance the reparative bioactivity of BMSC-derived sEVs and used GelMA as an established matrix for their sustained local delivery in diabetic wounds. SW-sEVs were isolated and characterized, and their effects on endothelial cell function and macrophage inflammatory phenotype were assessed *in vitro*. Proteomic analysis, together with validation of NF-κB-related proteins, was used to explore their potential anti-inflammatory and pro-angiogenic mechanisms. SW-sEVs were then incorporated into GelMA, and the resulting system was characterized for structure, mechanical properties, swelling, degradation, biocompatibility, and release behavior. Its effects on wound closure, angiogenesis, macrophage polarization, collagen deposition, and tissue remodeling were further evaluated in a diabetic rat wound model. This study establishes a sustained local delivery platform that combines enhanced sEV bioactivity with GelMA-mediated retention to promote diabetic wound repair (**Scheme 1**).

**Scheme 1:**
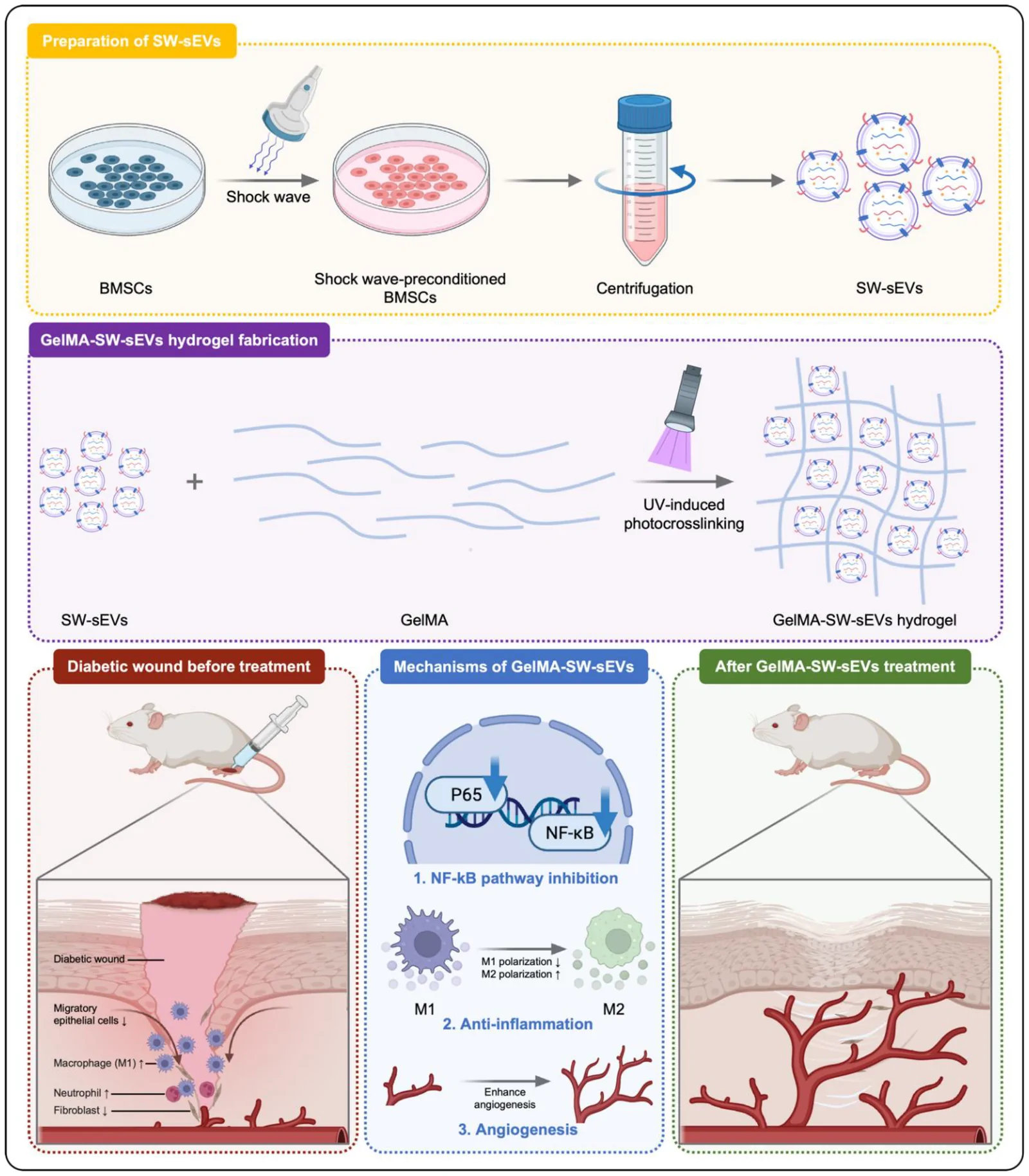
Schematic overview of the preparation, fabrication, and therapeutic mechanism of GelMA-SW-sEVs hydrogel for diabetic wound repair.

## 2 Methods

### 2.1 Cells and animals

BMSCs were isolated from the bone marrow of 3-week-old Sprague–Dawley (SD) rats and cultured in DMEM/F12 supplemented with 10% exosome-depleted fetal bovine serum and 1% penicillin/streptomycin (P/S). Human umbilical vein endothelial cells (HUVECs) and RAW264.7 macrophages were obtained from the Cell Bank of the Chinese Academy of Sciences and maintained in DMEM/F12 containing 10% fetal bovine serum and 1% P/S. All cells were cultured at 37 °C in a humidified incubator with 5% CO2. When cultures reached approximately 80% confluence, cells were passaged at a 1:3 ratio. During the experiments, all cells showed normal growth morphology, the culture medium remained clear, and no signs of contamination were observed. All cell-handling procedures were performed under aseptic conditions with strict measures to prevent cross-contamination.

All animal experiments were approved by the Ethics Committee of The First Hospital of Jilin University (Approval No. 20250414-02). Male SD rats aged 6–8 weeks and weighing 180–220 g, or aged 3–4 weeks and weighing 100–150 g, were purchased from Jilin Qianhe Model Biotechnology Co., Ltd. (Changchun, China). Animals were housed under specific pathogen-free conditions. All surgical procedures were performed under isoflurane anesthesia (RWD, China).

### 2.2 Optimization of shock wave preconditioning and sEV treatment conditions

Third- to sixth-passage BMSCs (P3–P6) were resuspended in DMEM/F12 complete medium containing 10% fetal bovine serum and 1% P/S at a density of 5 × 10^4 cells/mL, and 1 mL of cell suspension was transferred into sterile 1.5-mL microcentrifuge tubes. SW stimulation was delivered using a radial shock wave device equipped with a 10-mm applicator at a fixed frequency of 5 Hz. Four pressure levels (1, 2, 3, and 4 bar) and four pulse numbers (300, 600, 900, and 1200 pulses) were initially evaluated based on BMSC viability (24). The applicator was coupled to the tubes using sterile ultrasound coupling gel, and control cells underwent the same procedures without SW exposure.

To determine the working concentration of sEVs, HUVECs were seeded in 96-well plates at 1 × 10^3 cells/well and treated with 0, 20, 40, or 80 μg/mL sEVs or SW-sEVs for 24 h. Cell viability was assessed using the CCK-8 assay, with absorbance measured at 450 nm after a 2-h incubation with CCK-8 reagent. For functional optimization of SW preconditioning, sEVs derived from BMSCs exposed to different SW conditions were applied to HUVECs at 40 μg/mL, and HUVEC viability was evaluated by CCK-8 assay. Selected conditions showing favorable effects on HUVEC viability were further assessed using the tube formation assay. The final SW condition was selected based on both BMSC viability and the pro-angiogenic activity of the resulting sEVs.

### 2.3 Isolation, purification, and characterization of sEVs

sEVs were isolated by differential ultracentrifugation. Briefly, cell culture supernatants were collected and centrifuged at 500 × g for 5 min to remove cells, followed by filtration through a 0.22-μm filter to eliminate residual cells and large particulate debris. The filtrate was then centrifuged at 2,000 × g for 30 min and subsequently at 10,000 × g for 30 min to further remove cellular debris and large vesicles. The clarified supernatant was transferred into ultracentrifuge tubes and centrifuged at 100,000 × g for 70 min using a floor-standing ultracentrifuge. After the supernatant was discarded, the pellet was gently resuspended in 10 mL PBS and centrifuged again at 100,000 × g for 70 min to further purify the sEVs. All centrifugation steps were performed at 4 °C. The final sEV pellet was resuspended in prechilled sterile PBS and stored at -80 °C until further use.

The morphology of sEVs was examined by transmission electron microscopy (TEM; Hitachi HT-7800, Japan). Particle size distribution and concentration were determined using nanoparticle tracking analysis (NTA; ZetaView x30, Germany). The sEV-associated markers CD63, CD9, and TSG101 and the negative marker Calnexin were detected by Western blotting using primary antibodies against CD63, CD9, TSG101, and Calnexin (all 1:1000; Proteintech, Wuhan, China). Parental BMSC lysates were included as a cellular control for Calnexin. For protein extraction, sEV samples were lysed in RIPA buffer supplemented with protease inhibitors, and protein concentrations were measured using a BCA protein assay kit (Beyotime, Shanghai, China).

### 2.4 Cellular uptake of sEVs

sEVs were labeled using a PKH26 fluorescent labeling kit (Solarbio, Beijing, China). PKH26 dye was diluted 10-fold in Diluent C to prepare the working solution. After protein quantification by BCA assay, an appropriate amount of sEVs was mixed with the PKH26 working solution, vortexed for 1 min, and incubated in the dark for 10 min. To remove unbound dye, the labeled sEVs were purified by ultracentrifugation at 100,000 × g and 4 °C for 70 min, followed by resuspension in sterile PBS for subsequent use.

PKH26-labeled sEVs were incubated with HUVECs or RAW264.7 macrophages in the dark for 3 h. After incubation, cells were fixed with 4% paraformaldehyde, permeabilized with 0.1% Triton X-100, and stained for F-actin using phalloidin (1:200; Beyotime, Shanghai, China). Nuclei were counterstained with DAPI (1 μg/mL; Solarbio, Beijing, China). sEV internalization was visualized using a confocal laser scanning microscope (Nikon AXR, Japan).

### 2.5 Scratch assay

A scratch assay was performed to evaluate the effect of sEVs on HUVEC migration. HUVECs were seeded into 6-well plates at a density of 2 × 10^5 cells/well and cultured until they formed a confluent monolayer. A linear scratch was then gently created in the center of the monolayer using a sterile 200-μL pipette tip, and the wells were washed three times with PBS to remove detached cells. The medium was subsequently replaced with fresh medium containing 40 μg/mL sEVs. Images were acquired at 0, 12, and 24 h after scratching. Changes in the scratch area were quantified using ImageJ software and normalized to the initial wound area.

### 2.6 Transwell assay

HUVEC migration was assessed using 12-well Transwell chambers with 8-μm pore-size membranes (Corning, USA). HUVECs were seeded into the upper chamber in serum-free medium, whereas the lower chamber was filled with complete medium containing 40 μg/mL sEVs. After incubation for 24 h at 37 °C in 5% CO2, non-migrated cells remaining on the upper surface of the membrane were gently removed. Cells that migrated to the lower surface were fixed with 4% paraformaldehyde for 10 min and stained with 0.1% crystal violet. Migrated cells were then imaged and counted under a light microscope, and quantitative analysis was performed using ImageJ software.

### 2.7 Tube formation assay

The effect of sEVs on the angiogenic capacity of HUVECs was evaluated using a Matrigel-based tube formation assay. Briefly, 24-well plates were pre-coated with Matrigel (Corning), and HUVECs were seeded at a density of 2.0 × 10^4 cells/well. Cells were then cultured in medium containing 10% fetal bovine serum and 40 μg/mL sEVs. After incubation for 3 h at 37 °C in 5% CO2, tube-like structures were imaged under a light microscope. The number of nodes and total tube length were quantified using ImageJ software.

### 2.8 Cell immunofluorescence staining

Cells were fixed with 4% paraformaldehyde for 10 min, permeabilized with 0.1% Triton X-100 for 10 min, and blocked with PBS containing 5% bovine serum albumin (BSA) for 1 h. The cells were then incubated overnight at 4 °C with primary antibodies against VEGFA, CD31, Arg-1, CD206, iNOS, and CD86 (1:500; Proteintech). After washing with PBS, cells were incubated with the corresponding fluorophore-conjugated secondary antibodies (1:1000; Proteintech) for 1 h at room temperature in the dark. Nuclei were counterstained with DAPI for 5 min. Fluorescence images were acquired using a confocal laser scanning microscope (Nikon), and relative fluorescence intensity was quantified using ImageJ software.

### 2.9 Western blot analysis

Total proteins from cells, sEVs, and SW-sEVs were extracted using RIPA lysis buffer supplemented with protease and phosphatase inhibitors. Protein concentrations were determined using a BCA protein assay kit (Beyotime). Equal amounts of protein samples (10–20 μg) were separated by 10% SDS-PAGE (Coolaber, China) and transferred onto PVDF membranes (Millipore, Merck, Germany). After blocking with 5% BSA for 1 h, the membranes were incubated overnight at 4 °C with primary antibodies against CD9, CD63, TSG101, Calnexin, VEGFA, VEGFR2, iNOS, CD86, Arg-1, CD206, IL-1β, IL-6, TNF-α, β-actin, and α-tubulin (all 1:1000; Proteintech), as well as p65, p-p65, IκBα, and p-IκBα (all 1:1000; Selleck). After washing with TBST, the membranes were incubated with HRP-conjugated secondary antibodies (1:10,000; Proteintech) for 1 h at room temperature. Protein bands were visualized using an ECL chemiluminescent substrate and captured with a chemiluminescence imaging system. Band intensities were quantified by densitometric analysis using ImageJ software and normalized to the corresponding internal control protein.

### 2.10 Induction of polarization in RAW264.7 macrophages

RAW264.7 macrophages were cultured in complete DMEM containing 10% fetal bovine serum and 1% penicillin/streptomycin at 37 °C in a humidified atmosphere with 5% CO_2_. At approximately 80% confluence, cells were harvested and seeded in 12- or 6-well plates. The Control group was pretreated with an equivalent volume of PBS, whereas the sEVs and SW-sEVs groups were pretreated with 40 μg/mL sEVs or SW-sEVs, respectively, for 2 h. M1-like inflammatory activation was then induced by adding lipopolysaccharide (LPS; 1 μg/mL; Solarbio), followed by incubation for an additional 24 h in DMEM containing 10% exosome-depleted fetal bovine serum and 1% penicillin/streptomycin. Macrophage-associated phenotypic changes were evaluated by assessing the expression of the M1-like markers iNOS and CD86, the M2-like markers Arg-1 and CD206, and the inflammatory cytokines IL-1β, IL-6, and TNF-α.

### 2.11 Preparation and characterization of GelMA hydrogels

GelMA was purchased from Life Engineering Co., Ltd. (Suzhou, China). To identify the optimal hydrogel concentration for SW-sEV delivery, GelMA was dissolved in sterile PBS to prepare 5%, 10%, and 15% GelMA solutions. After complete dissolution, the solutions were sterilized by filtration through a 0.22-μm membrane. GelMA solutions at different concentrations were then cast into molds and photocrosslinked under UV irradiation for 1 min to obtain hydrogels with varying GelMA concentrations.

To examine the microscopic pore structure, photocrosslinked GelMA hydrogels were prefrozen at -80 °C and subsequently lyophilized. After gold sputter coating, the internal morphology of the freeze-dried samples was observed using scanning electron microscopy (SEM; ZEISS Gemini 300, Germany). The average pore size of GelMA hydrogels at different concentrations was measured from SEM images using ImageJ software. Porosity was calculated using the threshold analysis function in ImageJ.

To evaluate swelling behavior, GelMA hydrogel samples with a diameter of 10 mm and thickness of 5 mm were placed in sealed centrifuge tubes containing PBS (pH 7.4) and incubated at 37 °C. At predetermined time points, samples were removed, and excess surface PBS was gently blotted with filter paper before weighing. The swelling ratio was calculated as follows:


$$Swelling\;ratio(\%)=\frac{W_{t}-W_{0}}{W_{0}}\times 100\%$$

where W_0_ represents the initial hydrogel mass, and W_t_ represents the swollen hydrogel mass at each time point. This collagenase-mediated degradation assay was performed independently from the SW-sEV release experiment described below, which was conducted in collagenase-free PBS.

To assess *in vitro* degradation, fully swollen GelMA hydrogel samples were immersed in PBS containing type II collagenase (0.02 U/mL) and incubated at 37 °C. At predetermined time points, samples were removed, gently blotted with filter paper to remove excess surface liquid, and weighed. The residual mass and degradation rate were calculated using the following equations:


$$Residual\;mass(\%)=\frac{W_{t}}{W_{0}}\times 100\%$$

$$Degradation\;rate(\%)=\frac{W_{0}-W_{t}}{W_{0}}\times 100\%$$

where W_0_ represents the initial hydrogel mass, and W_t_ represents the residual mass after degradation for the indicated time.

To compare the mechanical properties of GelMA hydrogels at different concentrations, rectangular hydrogel specimens measuring 50 mm × 10 mm × 3 mm were prepared. Tensile testing was performed using a universal testing machine (CMT6103, China) at a crosshead speed of 2 mm/min. Stress–strain curves were recorded for each group, and the elongation at break and ultimate tensile strength were calculated.

To evaluate the cytocompatibility of GelMA hydrogels at different concentrations, cell viability was assessed using the CCK-8 assay. Cells were co-cultured with GelMA hydrogels or treated with hydrogel extracts prepared from different GelMA concentrations. At predetermined time points, CCK-8 working solution was added, and absorbance at 450 nm was measured to assess the effects of the hydrogels on cell viability.

### 2.12 Preparation and characterization of GelMA-SW-sEVs

To investigate the release profile of SW-sEVs from GelMA hydrogels, SW-sEVs (40 μg/mL) were homogeneously mixed with 200 μL of GelMA solutions at different concentrations to prepare 5%, 10%, and 15% GelMA-SW-sEVs hydrogels, corresponding to a nominal SW-sEV input of 8 μg per hydrogel construct. The mixtures were subsequently photocrosslinked under the conditions described above to form GelMA-SW-sEVs hydrogels. The GelMA-SW-sEVs hydrogels were then placed in 24-well plates containing 1 mL PBS per well and incubated at 37 °C. The supernatant was collected daily for 20 consecutive days, and the concentration of released SW-sEVs was determined using a BCA protein assay kit (Solarbio, Beijing, China). To assess whether the applied photocrosslinking conditions caused evident structural alterations to the incorporated SW-sEVs, vesicles released from the GelMA hydrogel were collected and examined by TEM.

To evaluate cellular uptake of SW-sEV released from GelMA-SW-sEVs hydrogels, SW-sEVs were first labeled with PKH26 and then incorporated into 15% GelMA hydrogels using the same preparation and photocrosslinking procedure described above. A co-culture system was established using Transwell chambers with 8-μm pore-size membranes (Corning). HUVECs or RAW264.7 macrophages were seeded in the lower chamber at a density of 4 × 10^4 cells/well, whereas GelMA-SW-sEVs hydrogels were placed in the upper chamber. After co-culture for 24, 48, or 72 h, HUVECs were stained with phalloidin to visualize the cytoskeleton and counterstained with DAPI to label nuclei. Fluorescence signals were visualized using a confocal laser scanning microscope. PKH26 fluorescence intensity was quantified using ImageJ under identical imaging and analysis settings and normalized to the corresponding fluorescence intensity at 24 h. Three independent experiments were performed.

To evaluate the *in vivo* retention of SW-sEVs after local administration, PKH26-labeled SW-sEVs were administered either in free form or encapsulated within 15% GelMA hydrogel. Fluorescence signals at the wound site were longitudinally monitored on days 1, 3, 5, and 7 using an *in vivo* fluorescence imaging system. Images were acquired using consistent imaging parameters across groups and time points. The fluorescence intensity within the wound-site region of interest was quantified, and relative fluorescence retention was normalized to the fluorescence intensity measured on day 1. Three rats were analyzed per group.

### 2.13 Animal experiments

Male SD rats were fasted for 12 h and then intraperitoneally injected with streptozotocin (STZ; 50 mg/kg) dissolved in 0.1 M citrate buffer (pH 4.5) to establish the diabetic model. After 1 week of high-fat diet feeding, random blood glucose levels were measured using tail vein blood samples. Rats with random blood glucose levels ≥16.7 mmol/L were considered successfully diabetic and were included for subsequent diabetic wound establishment. Anesthesia was induced and maintained with isoflurane (RWD, China). After adequate anesthesia was achieved, a 5-mm-diameter full-thickness skin defect was created on the dorsal aspect of the hind paw using a circular skin biopsy punch.

The rats were randomly divided into four groups (n = 6 per group): PBS, GelMA, GelMA-sEVs, and GelMA-SW-sEVs. After wound creation, 100 μL of GelMA, GelMA-sEVs (40 μg/mL), or GelMA-SW-sEVs (40 μg/mL) was applied to the wound surface and photocrosslinked *in situ* under UV irradiation for 20 s. Wound changes were recorded using a digital camera under fixed imaging conditions on days 1, 3, 5, 7, 10, and 14 after injury. Wound margins were traced using ImageJ software to quantify wound area and calculate the wound closure rate.

### 2.14 Histological examination

Wound tissues from different treatment groups were fixed in 4% paraformaldehyde for at least 24 h, dehydrated, paraffin-embedded, and sectioned at a thickness of 4 μm. Sections were stained with hematoxylin and eosin (H&E) or Masson’s trichrome staining kits (Solarbio, Beijing, China) and imaged using a VS200 whole-slide scanning system (Olympus, Japan). For preliminary *in vivo* biosafety assessment, the heart, liver, spleen, lung, and kidney were collected from rats in the Control, GelMA, GelMA-sEVs, and GelMA-SW-sEVs groups at the end of the treatment period and processed using the same fixation, embedding, and sectioning procedures, followed by H&E staining.

### 2.15 Immunohistochemistry and immunofluorescence staining of wound tissues

After paraffin-embedded tissue sections were deparaffinized and rehydrated, they were placed in Tris-EDTA antigen retrieval buffer (pH 9.0) and heated at 95 °C for 20 minutes. After cooling to room temperature, the sections were incubated in a peroxidase blocking solution for 10 minutes. Subsequently, the sections were permeabilized with PBS containing 0.1% Triton X-100 for 10 min and blocked with PBS containing 5% BSA at room temperature for 1 h.

The sections were incubated overnight at 4 °C with primary antibodies against VEGFA, Col1, and Col3 (1:1000), CD31 (1:5000; Proteintech), CD206, and iNOS (1:200; ServiceBio, Wuhan, China). For immunofluorescence staining, sections were washed with PBS, incubated with the corresponding fluorescently labeled secondary antibodies (1:1000; Proteintech) for 1 h at room temperature in the dark, and counterstained with DAPI for 5 min. For immunohistochemical staining, sections were washed with PBS, incubated with HRP-labeled secondary antibodies (1:500; Proteintech) for 1 h at room temperature, and developed with DAB solution for 1–5 min. Finally, sections were counterstained, dehydrated, cleared, mounted, and imaged using a VS200 whole-slide scanning system (Olympus).

### 2.16 Proteomic analysis of sEVs and SW-sEVs

Proteomic analysis of sEVs and SW-sEVs (n = 3 per group) was performed by Shanghai Genomics (Shanghai, China). Briefly, total vesicular proteins were extracted using lysis buffer containing 8 M urea, 1 mM PMSF, and 2 mM EDTA. Samples were sonicated on ice for 5 min and centrifuged at 15,000 × g for 10 min at 4 °C. The supernatant was collected, and protein concentration was determined using the BCA assay. For each sample, 100 μg of protein was reduced with 5 mM DTT at 37 °C for 45 min and alkylated with 11 mM iodoacetamide for 15 min at room temperature in the dark. The protein samples were then digested overnight at 37 °C with trypsin (Promega, V5280) in 25 mM NH_4_HCO_3_ buffer. The resulting peptides were desalted using C18 columns and quantified with a Pierce Peptide Quantification Kit (Thermo Fisher Scientific, USA).

Peptides were separated using a NanoElute UHPLC system equipped with a C18 analytical column (15 cm × 75 μm, 1.6 μm; IonOpticks, Australia) and analyzed on a timsTOF Pro2 mass spectrometer (Bruker Daltonics, Germany) in ddaPASEF and diaPASEF acquisition modes. DIA data were processed using DIA-NN software (v1.8.1) in library-free mode and searched against the UniProt rat proteome database containing 47,930 entries. Precursor ion- and protein-level identifications were both controlled at a false discovery rate (FDR) of 1%.

### 2.17 Statistical analysis

All statistical analyses were performed using GraphPad Prism 10. Data are presented as the mean ± standard deviation (SD). For *in vitro* experiments, n represents independent biological experiments, whereas for animal experiments, n represents individual animals. Comparisons among multiple groups were performed using one-way or two-way ANOVA, as appropriate. Longitudinal measurements from the same animals were analyzed using two-way repeated-measures ANOVA. A p value < 0.05 was considered statistically significant.

## 3 Results

### 3.1 Characterization and uptake of sEVs and SW-sEVs

To optimize SW pretreatment, we first evaluated the effects of different pressure intensities and pulse numbers on BMSC viability. Although several SW conditions maintained comparable BMSC viability, BMSC viability alone was insufficient to determine the optimal stimulation condition (**Supplementary Figure S1A**). We therefore further assessed the biological activity of sEVs derived from BMSCs exposed to different SW conditions. Functional screening showed that sEVs generated under 3 bar and 300 pulses produced the most favorable effect on HUVEC viability among the tested conditions **(Figure S1C**). This condition also showed strong pro-angiogenic activity in the HUVEC tube formation assay, as evidenced by increased total tube length (**Supplementary Figure S2**). Based on the combined evaluation of BMSC viability and the functional activity of the resulting sEVs, 3 bar, 300 pulses, and 5 Hz was selected for subsequent experiments.

sEVs and SW-sEVs were isolated from the culture supernatants of untreated and SW-pretreated BMSCs, respectively, and characterized by transmission electron microscopy (TEM), nanoparticle tracking analysis (NTA), and Western blotting. TEM revealed that both vesicle populations exhibited the characteristic nanoscale cup-shaped or spherical membrane morphology (**Figure 1A**). NTA showed comparable size distributions, with mean particle diameters of 125.8 nm for sEVs and 127.9 nm for SW-sEVs. The corresponding particle concentrations were 1.0 × 10^10 and 1.6 × 10^10 particles/mL, respectively (**Figure 1B**). Western blotting further confirmed the expression of the sEV-associated markers CD9, CD63, and TSG101 in both sEVs and SW-sEVs, whereas Calnexin was detected in parental BMSCs but was absent from both sEV preparations (**Figure 1C**).

**Figure 1:**
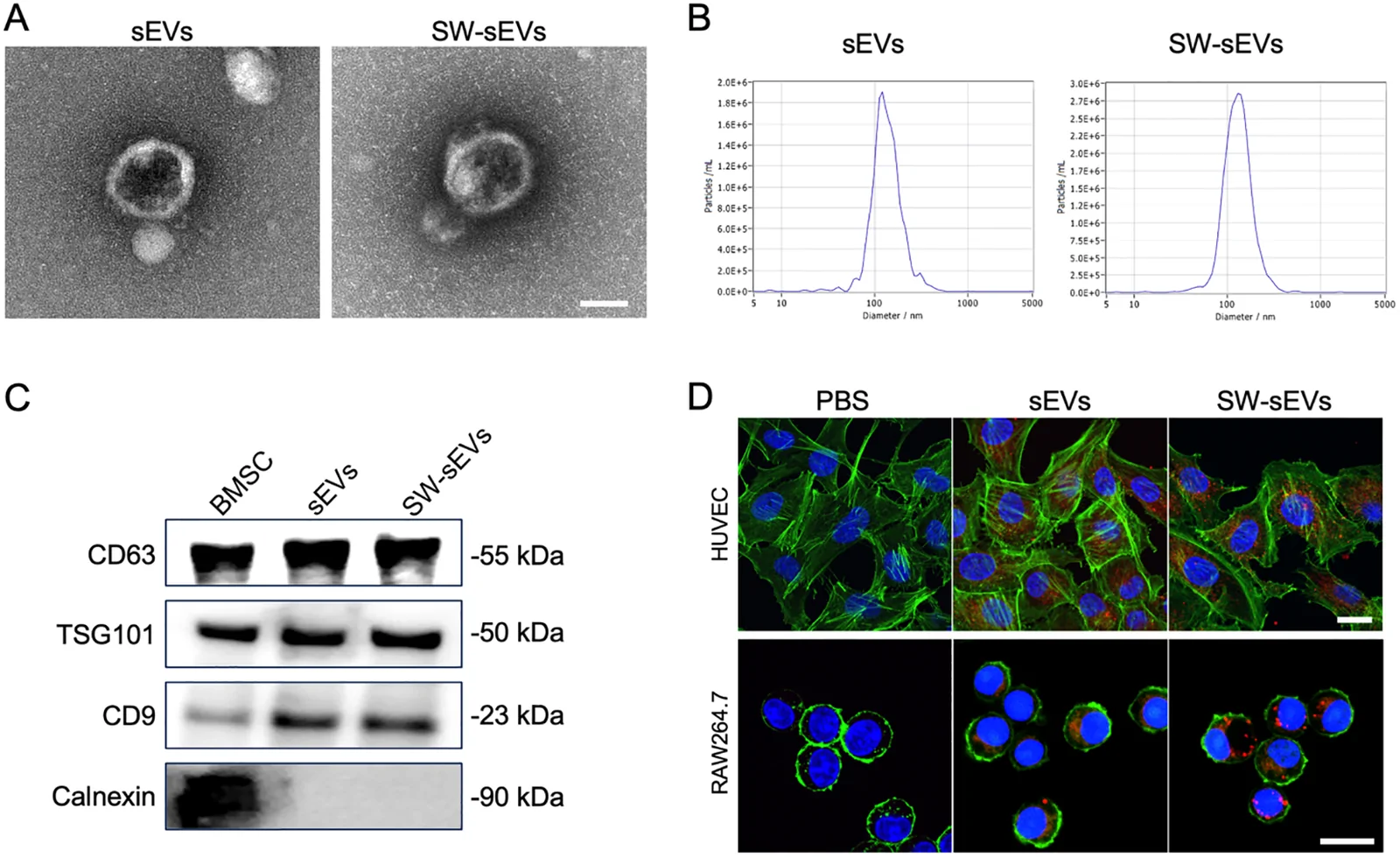
Characterization and cellular uptake of BMSC-derived sEVs and SW-sEVs. **(A)** Representative transmission electron microscopy (TEM) images showing the morphology of sEVs derived from untreated BMSCs (sEVs) and shock wave-preconditioned BMSCs (SW-sEVs). Scale bar, 50 nm. **(B)** Nanoparticle tracking analysis (NTA) showing the particle size distributions of sEVs and SW-sEVs. **(C)** Western blot analysis of the sEV-associated markers CD63, TSG101, and CD9 and the negative marker Calnexin in parental BMSCs, sEVs, and SW-sEVs. Calnexin was detected in BMSCs but was absent from both sEV preparations. **(D)** Representative confocal fluorescence images showing the uptake of PKH26-labeled sEVs and SW-sEVs by HUVECs and RAW264.7 macrophages. PBS-treated cells served as controls. PKH26-labeled sEVs are shown in red, the actin cytoskeleton in green, and nuclei in blue. Scale bar, 20 μm. Data are representative of three independent experiments (n = 3).

To assess cellular internalization, PKH26-labeled sEVs and SW-sEVs were separately incubated with HUVECs and RAW264.7 cells for 3 h. Confocal microscopy detected PKH26-positive red fluorescence in both cell types, predominantly within the perinuclear cytoplasm (**Figure 1D**). These findings demonstrate that both sEVs and SW-sEVs can be internalized by endothelial cells and macrophages, supporting subsequent evaluation of their proangiogenic and macrophage-modulatory activities.

### 3.2 SW-sEVs enhance endothelial angiogenic activity

To evaluate the effects of sEVs and SW-sEVs on endothelial cell function, HUVEC proliferation was first assessed using the CCK-8 assay after treatment with different vesicle concentrations. Within the tested range of 0–80 μg/mL, both sEVs and SW-sEVs promoted HUVEC proliferation in a concentration-dependent manner. This effect reached a plateau at concentrations above 40 μg/mL, with no further significant increase at higher doses. At equivalent concentrations, SW-sEVs exerted a stronger proliferative effect than native sEVs (**Supplementary Figure S1B**). Considering both biological efficacy and vesicle yield, 40 μg/mL was selected as the working concentration for subsequent experiments.

The effects of sEVs and SW-sEVs on HUVEC migration were then evaluated using scratch and Transwell assays. In the scratch assay, both vesicle treatments enhanced HUVEC migration into the wound area compared with the control group. In contrast, SW-sEVs produced the greatest reduction in the residual scratch area, indicating a stronger promigratory effect (**Figures 2A, B**). Consistently, the Transwell assay showed that both sEVs and SW-sEVs increased cell migration across the membrane, with significantly more cells migrating in the SW-sEVs group (**Figures 2C, D**). Together, these findings demonstrate that SW pretreatment enhances the promigratory activity of BMSC-derived sEVs.

**Figure 2:**
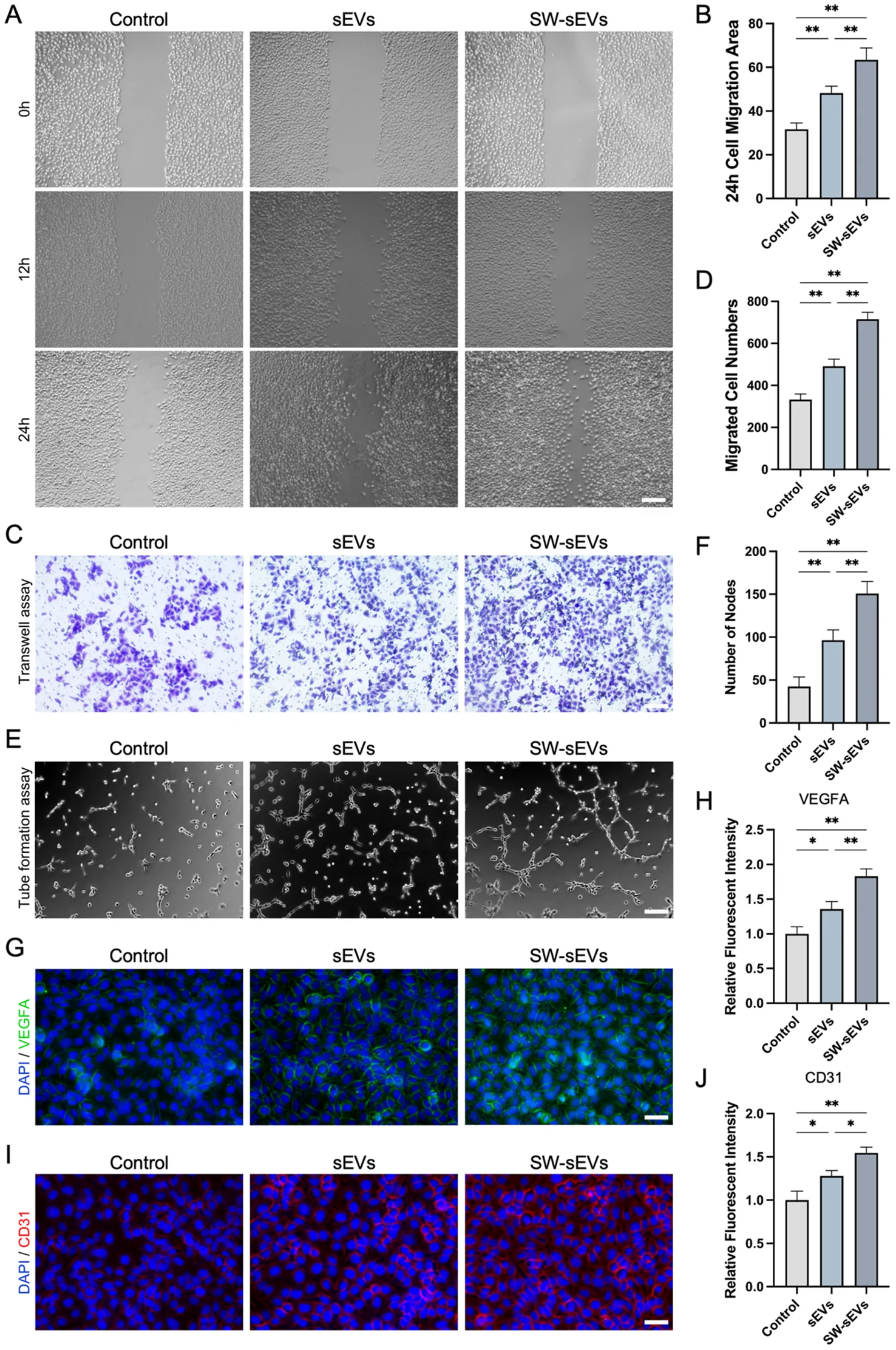
SW-sEVs enhance endothelial angiogenic activity. **(A)** Representative scratch assay images showing HUVEC migration in the Control, sEVs, and SW-sEVs groups at 0, 12, and 24 h. Scale bar, 100 μm. **(B)** Quantification of the migrated area at 24 h. **(C)** Representative Transwell migration images for the Control, sEVs, and SW-sEVs groups. Scale bar, 100 μm. **(D)** Quantification of migrated cells in the Transwell assay. **(E)** Representative tube formation images for the Control, sEVs, and SW-sEVs groups. Scale bar, 100 μm. **(F)** Quantification of tube formation by node number. **(G, I)** Representative immunofluorescence images of VEGFA and CD31 expression in the Control, sEVs, and SW-sEVs groups. Nuclei were counterstained with DAPI. Scale bar, 50 μm. **(H, J)** Quantification of the relative fluorescence intensities of VEGFA and CD31. Data are presented as mean ± SD (n = 3 independent experiments). *P < 0.05, **P < 0.01.

Because endothelial network formation is a key functional indicator of angiogenic capacity, the proangiogenic effects of the vesicles were further examined using a Matrigel tube formation assay. HUVECs in the control group formed only sparse and poorly connected tubular networks. Treatment with sEVs enhanced network formation, whereas SW-sEVs induced more extensive and interconnected tubular structures (**Figure 2E**). Quantitative analysis showed that SW-sEVs significantly increased the number of junctions (**Figure 2F**) and the total tube length compared with both the control and sEVs groups (**Supplementary Figure S3**). These results indicate that SW-sEVs markedly enhance the *in vitro* tube-forming capacity of HUVECs.

To further characterize the enhanced angiogenic phenotype, the expression of angiogenesis-related markers was examined. Immunofluorescence staining showed that sEV treatment increased VEGFA and CD31 signals in HUVECs compared with the control group, whereas SW-sEV treatment produced a further increase (**Figures 2G, I**). Quantitative analysis confirmed that the relative fluorescence intensities of VEGFA and CD31 were significantly higher in the SW-sEVs group than in the control and sEVs groups (**Figures 2H, J**). Western blotting further showed that SW-sEVs increased VEGFA and VEGFR2 protein expression in HUVECs (**Supplementary Figure S4**), consistent with the immunofluorescence and functional findings.

Collectively, these results demonstrate that SW-sEVs enhance HUVEC proliferation, migration, and tube formation while upregulating angiogenesis-related markers, including VEGFA, CD31, and VEGFR2. These findings indicate that SW pretreatment strengthens the proangiogenic activity of BMSC-derived sEVs.

### 3.3 SW-sEVs regulate macrophage polarization and inflammatory activation

Persistent inflammation and sustained M1-like macrophage activation are major contributors to impaired diabetic wound healing. Immunofluorescence staining was first performed to evaluate the effects of sEVs and SW-sEVs on the inflammatory phenotype of LPS-stimulated RAW264.7 macrophages. Compared with the LPS-treated control group, sEV treatment increased the fluorescence intensities of the M2-like macrophage markers Arg-1 and CD206, with a more pronounced increase observed following SW-sEV treatment (**Figures 3A, B, G, H**). Conversely, sEV treatment reduced the fluorescence signals of the M1-like macrophage markers iNOS and CD86, and these reductions were further enhanced by SW-sEVs (**Figures 3C, D, I, J**).

**Figure 3:**
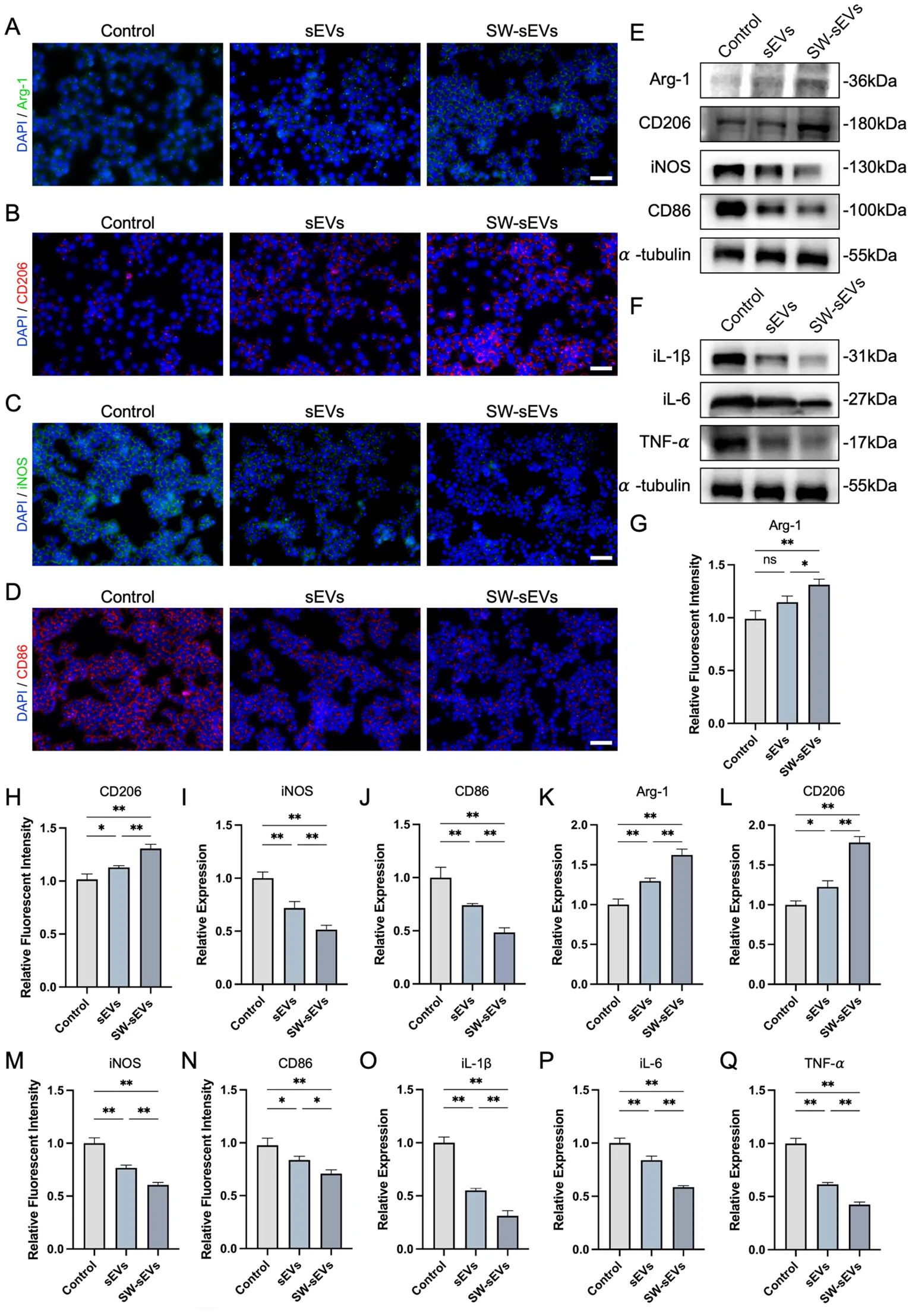
SW-sEVs regulate macrophage polarization and inflammatory activation. **(A–D)** Representative immunofluorescence images showing the expression of M2 macrophage markers Arg-1 and CD206, and M1 macrophage markers iNOS and CD86 in the Control, sEVs, and SW-sEVs groups. Nuclei were stained with DAPI. Scale bar, 50 μm. **(E, F)** Representative Western blot images showing the protein expression levels of M2-related markers Arg-1 and CD206, M1-related markers iNOS and CD86, and inflammatory cytokines IL-1β, IL-6, and TNF-α. α-tubulin was used as the loading control. **(G–J)** Quantification of immunofluorescence intensity for Arg-1, CD206, iNOS and CD86. **(K–Q)** Quantitative analysis of western blot bands for Arg-1, CD206, iNOS, CD86, IL-1β, IL-6, and TNF-α. Data are presented as the mean ± SD (n = 3 independent experiments). ns, not significant; *P < 0.05, **P < 0.01.

Western blotting confirmed these phenotypic changes. SW-sEV treatment increased Arg-1 and CD206 protein expression while decreasing iNOS and CD86 expression (**Figures 3E, K–N**). To further assess inflammatory activation, the expression of the representative proinflammatory cytokines IL-1β, IL-6, and TNF-α was examined. sEV treatment reduced the expression of all three cytokines, whereas SW-sEVs produced a more pronounced inhibitory effect (**Figures 3F, O–Q**).

Collectively, these findings indicate that SW pretreatment enhances the immunomodulatory activity of BMSC-derived sEVs by suppressing proinflammatory macrophage activation and promoting a shift toward a reparative macrophage phenotype.

### 3.4 Proteomic profiling reveals SW-induced remodeling of inflammatory pathways in sEVs

To determine whether SW pretreatment alters the protein composition of BMSC-derived sEVs and to explore the potential mechanisms by which SW-sEVs promote angiogenesis in endothelial cells and the polarization of M2-like macrophages, sEVs and SW-sEVs were collected 24 hours after SW pretreatment and subjected to proteomic analysis. Clustering heatmaps and volcano plots revealed significant differences in protein expression between the two groups. Compared with sEVs, 100 differentially expressed proteins were identified in SW-sEVs, including 48 upregulated and 52 downregulated proteins, while the majority of identified proteins showed no significant changes (**Figures 4A, B**). GO functional annotation and enrichment analysis revealed that the differentially expressed proteins were primarily involved in biological processes such as vasodilation, regulation of immune responses, and negative regulation of NF-κB transcriptional activity (**Figures 4C–E**). KEGG analysis further indicated that the differentially expressed proteins were enriched in the chemokine signaling pathway, the MAPK signaling pathway, and NF-κB-related inflammatory regulation modules (**Figure 4F**). To validate the inflammatory regulatory characteristics revealed by proteomics, key proteins of the NF-κB pathway were detected using Western blot analysis. Compared with the control group, sEVs treatment reduced the phosphorylation levels of p65 and IκBα, while SW-sEVs treatment further downregulated the expression of p-p65 and p-IκBα (**Figure 4G**). Quantitative analysis showed that the p-p65/p65 and p-IκBα/IκBα ratios in the SW-sEVs group were both significantly lower than those in the control and sEVs groups (**Figures 4H, I**). These results indicate that SW-sEVs can more effectively inhibit the activation of the NF-κB signaling pathway, consistent with their role in reducing pro-inflammatory cytokine expression and regulating macrophage polarization.

**Figure 4:**
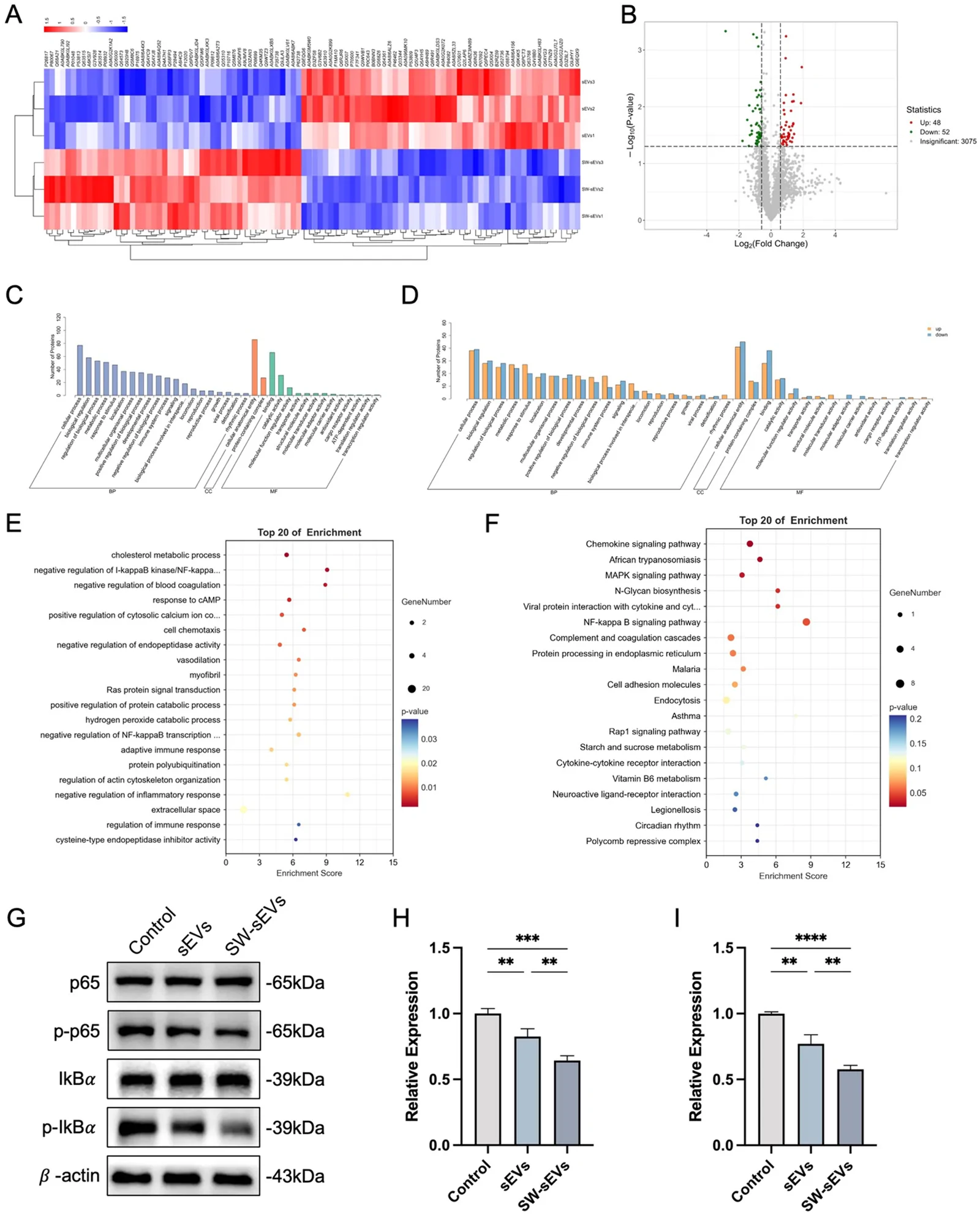
Proteomic profiling reveals SW-induced remodeling of inflammatory pathways in sEVs. **(A)** Hierarchical clustering heatmap of differentially expressed proteins between sEVs and SW-sEVs; red and blue indicate higher and lower expression, respectively. **(B)** Volcano plot comparing sEVs and SW-sEVs. Red, green, and gray dots denote significantly upregulated, significantly downregulated, and unchanged proteins, respectively. **(C)** Gene Ontology (GO) classification of differentially expressed proteins into biological process (BP), cellular component (CC), and molecular function (MF) categories. **(D)** Distribution of upregulated and downregulated proteins across major GO categories. **(E)** GO enrichment analysis of differentially expressed proteins. Dot size indicates protein number, and color denotes the corresponding P value. **(F)** KEGG pathway enrichment analysis of differentially expressed proteins. Dot size indicates protein number, and color denotes the corresponding P value. **(G)** Representative Western blots of total p65, phosphorylated p65 (p-p65), total IκBα, and phosphorylated IκBα (p-IκBα) in the Control, sEVs, and SW-sEVs groups. β-actin served as the loading control. **(H, I)** Quantification of the p-p65/p65 and p-IκBα/IκBα ratios, respectively. Data are presented as mean ± SD (n = 3 independent experiments). **P < 0.01, ***P < 0.001, and ****P < 0.0001.

### 3.5 Optimization and characterization of GelMA hydrogels for SW-sEV delivery

To develop a hydrogel carrier for the localized and sustained delivery of SW-sEVs, GelMA hydrogels were prepared at concentrations of 5%, 10%, and 15% and photocrosslinked under UV irradiation to form GelMA–SW-sEV composite systems (**Figure 5A**). Scanning electron microscopy (SEM) revealed interconnected porous networks in all GelMA formulations. As the GelMA concentration increased, the network became progressively denser, accompanied by reductions in porosity and mean pore diameter (**Figures 5B–D**).

**Figure 5:**
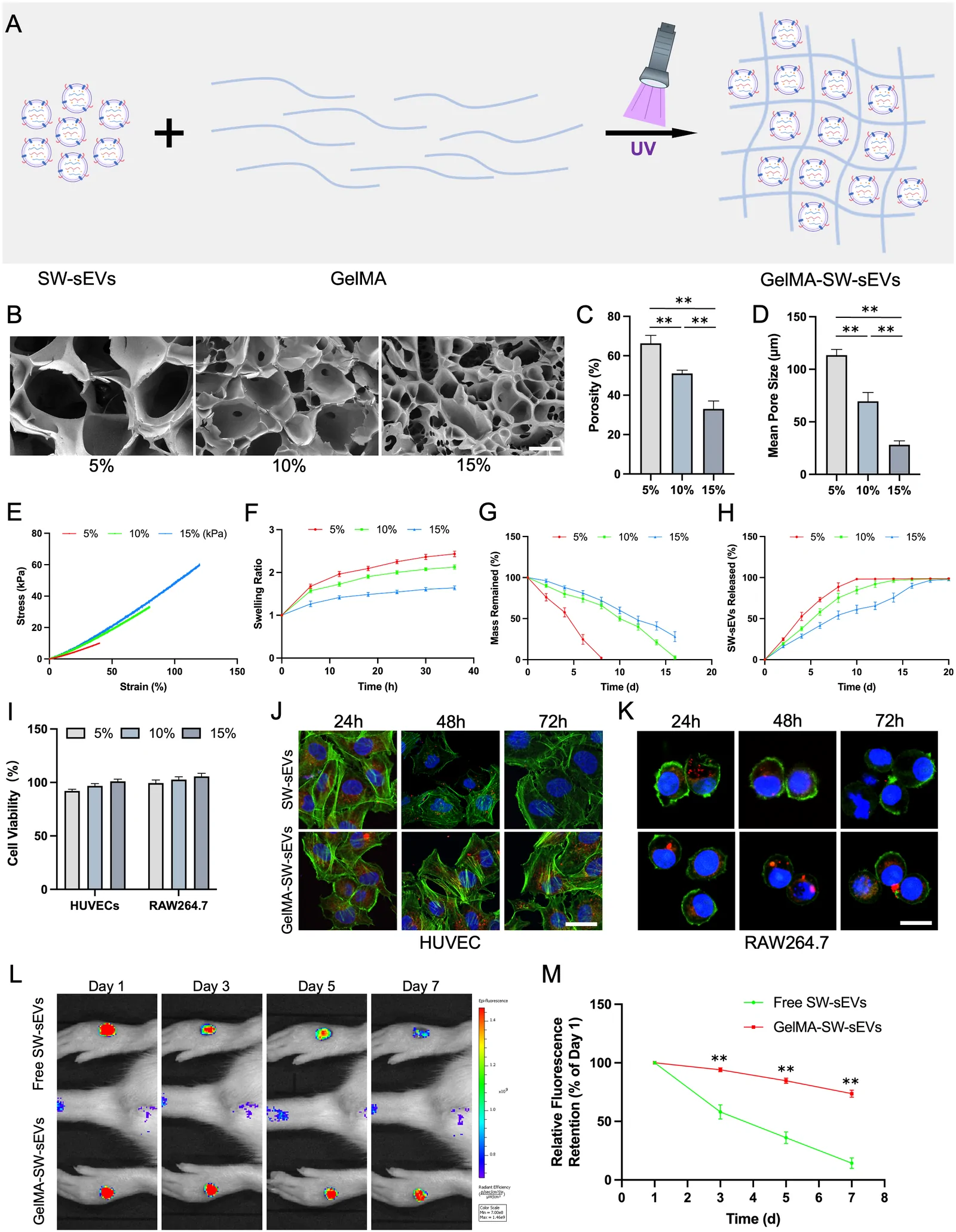
Fabrication, characterization, sustained release, and retention of GelMA-SW-sEVs. **(A)** Schematic illustration of the preparation of GelMA-SW-sEVs by encapsulating SW-sEVs within GelMA followed by UV-induced photocrosslinking. **(B)** Representative scanning electron microscopy (SEM) images of GelMA hydrogels at concentrations of 5%, 10%, and 15%, showing their porous microstructures. Scale bar, 200 μm. **(C, D)** Quantification of hydrogel porosity and mean pore size, respectively. **(E)** Representative stress–strain curves of 5%, 10%, and 15% GelMA hydrogels. **(F)** Swelling ratios of GelMA hydrogels over time. **(G)** *In vitro* collagenase-mediated degradation profiles of 5%, 10%, and 15% GelMA hydrogels, expressed as the percentage of remaining mass over time. **(H)** Cumulative release profiles of SW-sEVs from 5%, 10%, and 15% GelMA hydrogels in collagenase-free PBS at 37 °C. **(I)** Viability of HUVECs and RAW264.7 macrophages cultured with 5%, 10%, and 15% GelMA hydrogels. **(J, K)** Representative confocal fluorescence images showing the time-dependent cellular retention of PKH26-labeled SW-sEVs delivered as free SW-sEVs or GelMA-SW-sEVs in HUVECs and RAW264.7 macrophages, respectively, at 24, 48, and 72 h. PKH26-labeled SW-sEVs are shown in red, the actin cytoskeleton in green, and nuclei in blue. Scale bar, 20 μm. **(L)** Representative *in vivo* fluorescence images showing the retention of free SW-sEVs and GelMA-SW-sEVs at the administration site on days 1, 3, 5, and 7. **(M)** Quantification of relative fluorescence retention *in vivo*, normalized to the fluorescence intensity on day 1. For the *in vitro* quantitative analyses, data are presented as mean ± SD (n = 3 independent experiments). For the *in vivo* fluorescence retention analysis, n = 3 rats per group. ns, not significant; *P < 0.05 and **P < 0.01.

Mechanical testing showed that 15% GelMA exhibited the highest tensile strength (**Figure 5E**), together with the lowest water absorption (**Figure 5F**) and the slowest *in vitro* degradation rate, retaining the greatest residual mass at day 6 (**Figure 5G**). These findings are consistent with its denser network architecture and smaller pore size, indicating enhanced structural stability under enzymatic degradation conditions. Importantly, the degradation assay in **Figure 5G** was performed in collagenase-containing PBS and was independent of the subsequent SW-sEV release assay conducted in collagenase-free PBS; therefore, the degradation and release timelines should not be directly compared.

Release studies further showed that SW-sEVs were released more rapidly from 5% GelMA, whereas 15% GelMA markedly delayed vesicle release and extended the release period to approximately 20 days (**Figure 5H**). Despite the interconnected porous structure of GelMA, the cumulative release profile of 15% GelMA showed a gradual and prolonged release pattern, with no pronounced early burst evident at the measured time points. TEM examination of SW-sEVs released from photocrosslinked GelMA showed preservation of the characteristic vesicular morphology, with no evident structural impairment observed under the applied photocrosslinking conditions(**Supplementary Figure S5**).

CCK-8 assays showed that none of the three GelMA formulations significantly reduced the viability of HUVECs or RAW264.7 macrophages, indicating favorable cytocompatibility (**Figure 5I**). Fluorescence tracking was then used to assess the persistence of the SW-sEV-associated signal during GelMA-mediated delivery. Following treatment with GelMA-SW-sEVs, PKH26 fluorescence remained readily detectable in both HUVECs and RAW264.7 macrophages at 72 h, whereas the signal associated with free SW-sEVs progressively declined over time (**Figures 5J, K**). Quantitative analysis confirmed greater retention of PKH26-associated fluorescence in the GelMA-SW-sEVs group at later time points compared with the free SW-sEVs group (**Supplementary Figure S6**).

To further determine whether GelMA prolonged the local retention of SW-sEVs *in vivo*, longitudinal fluorescence imaging was performed after local administration of free PKH26-labeled SW-sEVs or GelMA-encapsulated PKH26-labeled SW-sEVs. The fluorescence signal in the free SW-sEV group progressively decreased over time, whereas stronger fluorescence was retained at the wound site in the GelMA-SW-sEV group through day 7 (**Figure 5L**). Quantitative analysis of the wound-site fluorescence further demonstrated greater retention of PKH26-associated fluorescence following GelMA-mediated delivery compared with free SW-sEV administration (**Figure 5M**). These findings provide *in vivo* evidence that GelMA prolongs the local persistence of SW-sEV-associated fluorescence after administration.

Collectively, 15% GelMA provided the most favorable overall performance in terms of pore architecture, mechanical strength, degradation resistance, cytocompatibility, and sustained SW-sEV release, and was therefore selected for subsequent experiments.

### 3.6 GelMA-SW-sEVs accelerate diabetic wound healing

To evaluate the *in vivo* therapeutic efficacy of GelMA-SW-sEVs, a full-thickness skin wound model was established in STZ-induced hyperglycemic diabetic rats. The animals were assigned to four groups: control, GelMA, GelMA-sEVs, and GelMA-SW-sEVs. Wound images were acquired on days 1, 3, 5, 7, 10, and 14 after treatment to longitudinally monitor the healing process (**Figure 6A**). Wound contour trajectories were further plotted at each time point to facilitate visual comparison among groups (**Figure 6B**). The GelMA-SW-sEVs group showed the greatest reduction in wound area, consistent with its superior healing performance. Quantitative analysis confirmed that both GelMA-sEVs and GelMA-SW-sEVs significantly accelerated wound closure, with the GelMA-SW-sEVs group exhibiting the fastest healing throughout the observation period and approaching complete closure by day 14 (**Figure 6C**).

**Figure 6:**
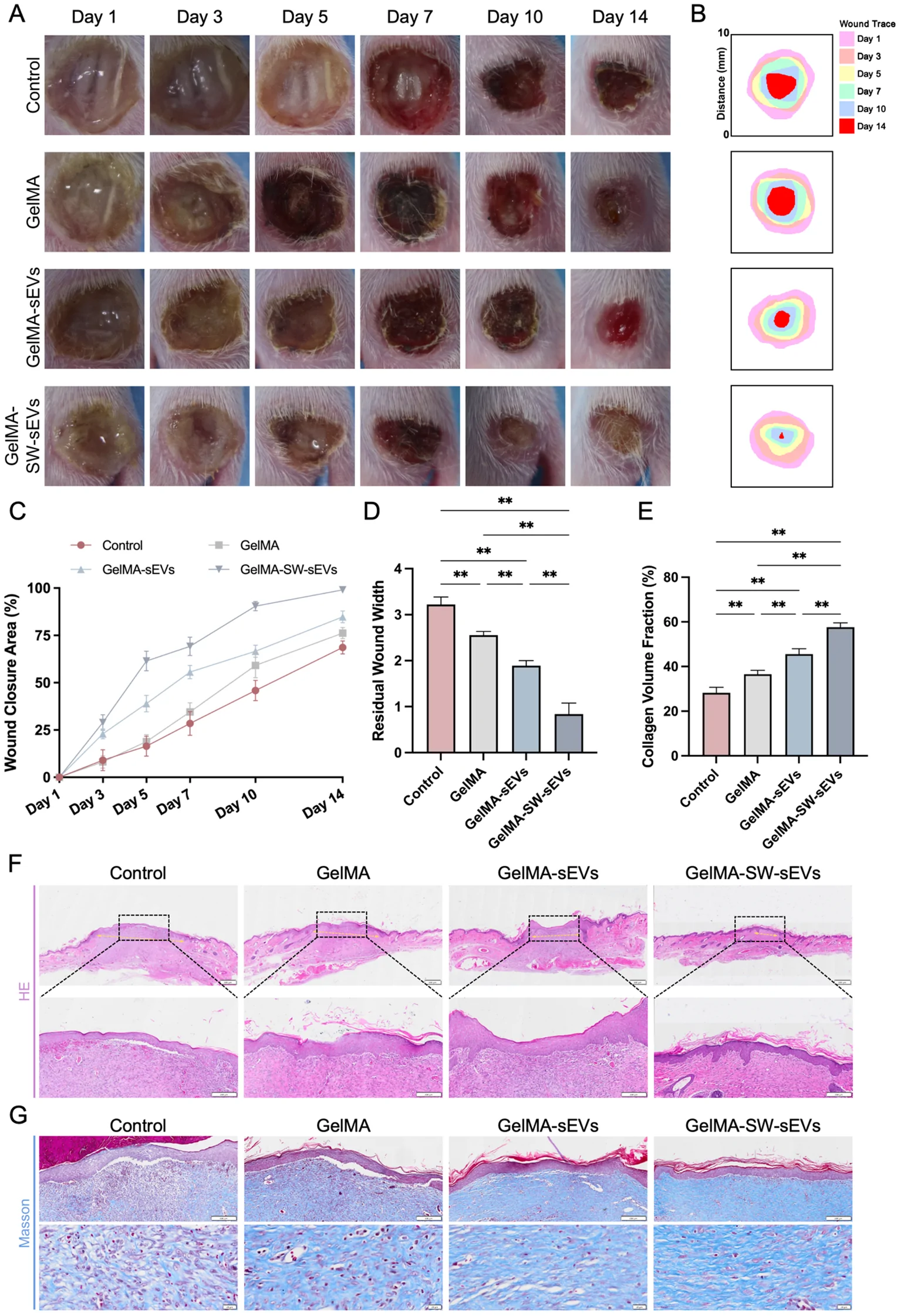
GelMA-SW-sEVs accelerate diabetic wound healing. **(A)** Representative gross images of wounds in the Control, GelMA, GelMA-sEVs, and GelMA-SW-sEVs groups on days 1, 3, 5, 7, 10, and 14 after treatment. **(B)** Representative wound tracings showing the progressive reduction in wound area in each group. **(C)** Quantification of wound closure over time. **(D)** Quantification of residual wound width on day 14. **(E)** Quantification of the collagen volume fraction in regenerated wound tissues. **(F)** Representative hematoxylin and eosin-stained sections of wound tissues on day 14. Boxed regions in the upper panels are shown at higher magnification below. Scale bars, 500 μm (upper) and 200 μm (lower). **(G)** Representative Masson’s trichrome-stained sections showing collagen deposition and tissue remodeling on day 14. Scale bars, 200 μm (upper) and 20 μm (lower). Data are presented as mean ± SD (n = 6 rats per group). **P < 0.01.

Histological repair and tissue regeneration were evaluated by hematoxylin and eosin (H&E) and Masson’s trichrome staining. H&E staining showed varying degrees of granulation tissue formation across all groups (**Figure 6F**). The mean granulation tissue widths in the control and GelMA groups were 3.22 and 2.55 mm, respectively, whereas the width decreased to 1.89 mm in the GelMA-sEVs group. In comparison, the GelMA-SW-sEVs group exhibited a more continuous regenerated epidermis, a more organized tissue architecture, and reduced inflammatory cell infiltration, together with the narrowest granulation tissue width of 0.84 mm (**Figure 6D**).

Masson’s trichrome staining revealed limited and loosely organized collagen deposition in the control group. Collagen accumulation increased in the GelMA group and was further enhanced in the GelMA-sEVs group, whereas the GelMA-SW-sEVs group displayed the most abundant and well-organized collagen fibers (**Figure 6G**). Quantitative analysis confirmed that the collagen volume fraction was significantly higher in the GelMA-SW-sEVs group than in the other groups (**Figure 6E**).

To further evaluate the preliminary *in vivo* biosafety of the treatments, H&E staining was performed on the heart, liver, spleen, lung, and kidney at the end of the treatment period. No obvious treatment-related histopathological abnormalities were observed in the GelMA, GelMA-sEVs, or GelMA-SW-sEVs groups compared with the control group (**Supplementary Figure S7**).

Collectively, these findings demonstrate that GelMA-SW-sEVs markedly accelerate diabetic wound healing and improve re-epithelialization, granulation tissue remodeling, and collagen deposition.

### 3.7 GelMA-SW-sEVs enhance angiogenesis, matrix remodeling, and immunoregulation

To further evaluate the effects of GelMA-SW-sEVs on angiogenesis, extracellular matrix remodeling, and the inflammatory microenvironment of diabetic wounds, immunohistochemical and immunofluorescence analyses were performed on wound tissues collected on day 14. VEGFA immunohistochemistry revealed only weak positive staining in the control and GelMA groups, whereas VEGFA expression was increased in the GelMA-sEVs group and was most pronounced and widely distributed in the GelMA-SW-sEVs group (**Figures 7A, G**). CD31 immunofluorescence showed a similar pattern, with weak signals in the control and GelMA groups, increased fluorescence in the GelMA-sEVs group, and the strongest signal in the GelMA-SW-sEVs group (**Figures 7B, H**).

**Figure 7:**
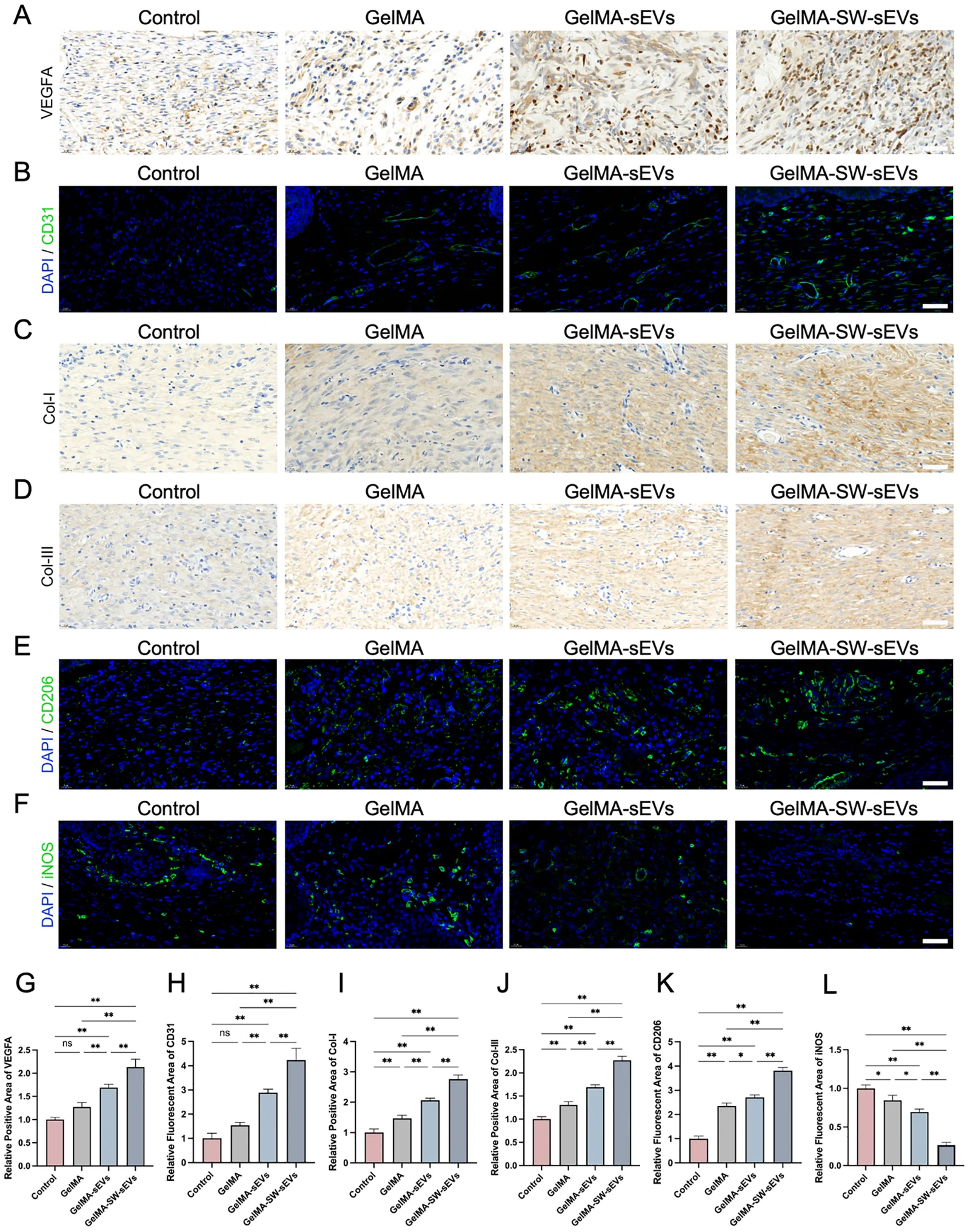
GelMA-SW-sEVs enhance angiogenesis, matrix remodeling, and immunoregulation. **(A)** Representative immunohistochemical images of VEGFA in wound tissues from the Control, GelMA, GelMA-sEVs, and GelMA-SW-sEVs groups. **(B)** Representative immunofluorescence images of CD31 in wound tissues. Nuclei were counterstained with DAPI. **(C, D)** Representative immunohistochemical images of COL1/COL3 in each group. **(E, F)** Representative immunofluorescence images of CD206-positive M2 macrophages and iNOS-positive M1 macrophages in wound tissues. Nuclei were counterstained with DAPI. **(G)** Quantification of the VEGFA-positive area. **(H)** Quantification of the CD31-positive fluorescence area. **(I, J)** Quantification of COL1/COL3-positive areas. **(K, L)** Quantification of CD206/iNOS-positive fluorescence areas. Scale bar, 20 μm. Data are presented as mean ± SD (n = 6 rats per group). ns, not significant; *P < 0.05, **P < 0.01.

To assess collagen deposition and extracellular matrix remodeling, COL1 and COL3 expression was further examined. Compared with the other groups, the GelMA-SW-sEVs group exhibited the strongest positive staining for both COL1 and COL3 (**Figures 7C, D, I, J**), indicating enhanced collagen deposition and matrix remodeling.

Macrophage-associated inflammatory phenotypes were subsequently evaluated using CD206 and iNOS as markers of M2-like and M1-like macrophages, respectively. The GelMA-SW-sEVs group exhibited the highest CD206 fluorescence intensity and the lowest iNOS signal among all groups (**Figures 7E, F, K, L**), suggesting suppression of the proinflammatory macrophage phenotype and promotion of a reparative macrophage response.

Collectively, these findings indicate that GelMA-SW-sEVs improve the diabetic wound microenvironment by enhancing VEGFA/CD31-associated angiogenic responses, promoting COL1/COL3-associated extracellular matrix remodeling, and shifting macrophage phenotypes toward a reparative state.

## 4 Discussion

Chronic non-healing DFUs are driven by a pathological wound microenvironment characterized by impaired angiogenesis, persistent inflammation, and dysregulated extracellular matrix remodeling (25, 26). Here, we developed a GelMA hydrogel-based platform for the local delivery of shockwave-preconditioned BMSC-derived small extracellular vesicles (SW-sEVs). Compared with native sEVs, SW-sEVs exerted stronger pro-angiogenic effects on HUVECs, more effectively suppressed NF-κB signaling, and produced greater immunomodulatory effects on RAW264.7 macrophages. These actions improved the wound inflammatory microenvironment and accelerated healing in diabetic rats.

Shock wave therapy is a physical intervention used to treat musculoskeletal disorders, including fracture nonunion, tendinopathies, and soft tissue injuries (27). Increasing evidence indicates that shock wave stimulation modulates stem cell behavior, angiogenesis, inflammatory responses, and extracellular vesicle secretion (28). Previous studies have combined transverse tibial bone transport or proximal tibial osteotomy with shock wave stimulation to activate endogenous repair and promote chronic wound healing; however, these approaches remain invasive (29). Here, BMSCs were preconditioned with shock waves *in vitro*, and the resulting SW-sEVs were collected for local wound treatment. This strategy preserved the ability of mechanical stimulation to enhance BMSC paracrine activity while avoiding trauma associated with bone manipulation (30). Importantly, the SW preconditioning parameters were selected based not only on BMSC viability but also on the functional bioactivity of the resulting sEVs, as assessed by HUVEC viability and tube formation. Mechanistically, shock waves may regulate BMSC activity through mechanotransduction, whereby transient mechanical stimuli are converted into intracellular biochemical signals (31). Previous studies have shown that shock wave stimulation can induce extracellular ATP release and activate purinergic receptor-dependent ERK1/2, p38 MAPK, and focal adhesion kinase signaling (32, 33). More recently, radial shock wave stimulation was reported to enhance sEV release from BMSCs through the ATP/P2X7R/p38 MAPK axis (17). These pathways may influence vesicle biogenesis, secretion, and cargo remodeling, thereby providing a plausible basis for the increased particle concentration and altered protein composition observed in SW-sEVs (34). Consistent with this possibility, SW-sEVs showed a higher particle concentration than native sEVs without substantial changes in vesicle morphology or size distribution, while proteomic analysis demonstrated selective remodeling of their protein cargo. Such changes may contribute to the enhanced pro-angiogenic and immunomodulatory effects of SW-sEVs observed in this study. However, the upstream signaling events responsible for SW-induced sEV remodeling were not directly investigated and therefore require further mechanistic validation.

A key pathological feature of DFUs is the failure of wounds to progress efficiently from the inflammatory phase to the proliferative and remodeling phases (35). Persistent M1-like macrophage activation causes prolonged release of TNF-α, IL-1β, IL-6, and other inflammatory mediators, thereby impairing endothelial function, inhibiting angiogenesis, disrupting fibroblast activity, and delaying collagen remodeling (36, 37). Previous studies have shown that MSC-derived exosomes promote macrophage phenotypic switching during early inflammation. Liu et al. reported that melatonin-preconditioned MSC exosomes promoted M1-to-M2 polarization by upregulating PTEN and suppressing AKT phosphorylation, reducing IL-1β, TNF-α, and iNOS while increasing IL-10 and Arg-1 (13). Similarly, Ti et al. showed that LPS-preconditioned MSC-derived sEVs delivered let-7b to regulate TLR4/NF-κB/STAT3/AKT signaling, suppress pro-inflammatory macrophages, and promote reparative polarization, thereby alleviating early chronic inflammation and accelerating diabetic wound healing more effectively than native MSC exosomes (38). These findings support the ability of MSC-sEV bioactive cargo to regulate macrophage polarization and cytokine secretion. Consistently, we found that SW-sEVs downregulated iNOS, CD86, IL-1β, IL-6, and TNF-α while upregulating Arg-1 and CD206 in RAW264.7 macrophages. In diabetic wound tissues, GelMA-SW-sEVs likewise reduced iNOS-positive signals and enhanced CD206-positive signals.

The pro-angiogenic activity of GelMA-SW-sEVs represents another major component of their therapeutic efficacy. Endothelial dysfunction and inadequate neovascularization are key contributors to delayed DFU healing (26). Previous biomaterial-based strategies have largely focused on restoring angiogenesis (39). Han et al. incorporated ADSC-derived exosomes into an oxygen-carrying nanobubble hydrogel, which alleviated hypoxia and enhanced exosome uptake, thereby improving endothelial function, neovascularization, and diabetic wound repair (40). Yu et al. preconditioned BMSCs with atorvastatin to enrich exosomal miR-221-3p and activate endothelial AKT/eNOS signaling, consequently enhancing migration, tube formation, and *in vivo* angiogenesis (41). In the present study, the GelMA-SW-sEV system combines the enhanced pro-angiogenic and immunomodulatory activities of SW-sEVs with GelMA-mediated local delivery, thereby targeting both inflammatory and angiogenic dysfunction in diabetic wounds.

From a mechanistic perspective, NF-κB signaling may be a central pathway through which SW-sEVs modulate macrophage inflammatory activation (42). Macrophage polarization is governed by multiple pathways, including PI3K/AKT, JAK/STAT, and NF-κB (42). Recent studies indicate that NF-κB and MAPK signaling remain persistently activated in the hyperglycemic, oxidative, and inflammatory milieu of diabetic wounds (43). Activation of the IκBα/p65 axis promotes p65 phosphorylation and nuclear translocation, drives pro-inflammatory gene transcription, and sustains the M1-like macrophage phenotype (43). In this study, proteomic enrichment analysis showed that proteins differentially expressed in SW-sEVs were closely associated with immune and inflammatory pathways. Western blotting further demonstrated that SW-sEVs reduced the p-p65/p65 and p-IκBα/IκBα ratios in macrophages, indicating suppression of NF-κB activation. These findings are consistent with recent studies of immunomodulatory hydrogels for diabetic ulcers, in which NF-κB inhibition was associated with reduced inflammatory mediator production, enhanced M2-like polarization, and improved wound healing (44). NF-κB suppression may also indirectly support angiogenesis and collagen remodeling, as excessive inflammatory signaling impairs endothelial function, destabilizes newly formed vessels, and disrupts extracellular matrix deposition (37). Thus, NF-κB signaling may represent a key mechanistic link among the anti-inflammatory, pro-angiogenic, and tissue-remodeling effects of GelMA-SW-sEVs.

GelMA served primarily as the local delivery matrix in this system. Free extracellular vesicles are prone to rapid clearance *in vivo*, which can limit their local therapeutic availability (45). Owing to its biocompatibility, degradability, and tunable network and mechanical properties, GelMA is widely used as a photocrosslinkable carrier for local biomolecule delivery (22). In our study, 15% GelMA exhibited higher tensile strength, lower swelling and degradation, and more prolonged SW-sEV release than lower-concentration formulations. Similarly, Zhao et al. reported that increasing GelMA concentration from 5% to 15% increased tensile strength from 4 ± 1 to 24 ± 2 kPa while reducing the swelling ratio from 1476 ± 28% to 567 ± 19% (46), supporting enhanced network stability at higher GelMA concentrations. Consistent with previous GelMA–EV systems (47, 48), our *in vivo* imaging further showed prolonged local retention of PKH26-associated fluorescence, supporting GelMA as a delivery matrix that extends SW-sEV availability while SW-sEVs provide the principal biological activity.

This study focused on diabetic wounds because hyperglycemia is a major pathological factor contributing to endothelial dysfunction, persistent inflammation, and impaired tissue repair. *In vivo*, diabetes was induced by STZ and confirmed by random blood glucose levels ≥16.7 mmol/L before wound creation, thereby establishing a hyperglycemic background for wound-healing assessment. However, this model represents a diabetic wound rather than the full clinical spectrum of DFUs, which are often complicated by ischemia, neuropathy, infection, biofilm formation, and recurrent mechanical stress. Moreover, the *in vitro* experiments did not directly model high-glucose conditions. Future studies should therefore validate SW-sEV activity under hyperglycemic conditions and in more clinically representative DFU models. Second, although proteomic analysis indicated that shock wave preconditioning altered vesicular proteins associated with immune and inflammatory regulation, the key functional proteins responsible for these effects remain unidentified and unvalidated. Future studies should integrate proteomic screening with loss- and gain-of-function experiments to determine whether specific vesicular cargo mediates NF-κB inhibition, macrophage polarization, and angiogenesis.

## 5 Conclusion

In summary, this study established a GelMA hydrogel-based platform for localized delivery of shock wave-preconditioned BMSC-derived sEVs to diabetic wounds. SW-sEVs exhibited enhanced reparative activity by promoting endothelial proliferation, migration, tube formation, and angiogenic marker expression, while suppressing M1-like macrophage activation and promoting an M2-like reparative phenotype. GelMA encapsulation enabled sustained local release and markedly accelerated wound closure, collagen deposition, neovascularization, and tissue remodeling. These findings support shock wave preconditioning as an effective strategy to enhance BMSC-sEV potency and establish GelMA-SW-sEVs as a cell-free regenerative platform for diabetic wound repair.

## Data Availability

The datasets presented in this study can be found in online repositories. The names of the repository/repositories and accession number(s) can be found below: IPX0018809000 (https://www.iprox.cn/page/project.html?id=IPX0018809000).
