# Supplementary material for: GelMA hydrogel enhances diabetic wound healing by delivering shock wave-preconditioned BMSC-derived small extracellular vesicles
Source: Front Immunol. 2026 Oct 28;17:1971302. doi: 10.3389/fimmu.2026.1971302 (PMC13751358; doi:10.3389/fimmu.2026.1971302)
Supplement: Supplementary file 1 [file SupplementaryFile1.docx]

Supplementary Material

**Supplemental information for**

GelMA Hydrogel Enhances Diabetic Wound Healing by Delivering Shock Wave-Preconditioned BMSC-Derived Small Extracellular Vesicles

Rongqi Zhou^1^, Xiaoping Xie^2^, Xudong Li^1^, Songqi Bi^1^, Zhixing Yan^1^, Xiaoyang Zhou^1^, Fanxu Wang^1^, Tiecheng Yu^1*^

^1^Department of Orthopedics, Orthopedics Center, The First Hospital of Jilin University, Jilin University, Changchun, Jilin, China.

^2^Department of Sports Medicine, Hubei Sports Medicine Center, Wuhan Fourth Hospital, Wuhan, China.

# 1 Supplementary Figures

**Figure S1. Optimization of shock wave preconditioning parameters based on BMSC viability and the effects of BMSC-derived sEVs on HUVEC viability.** (A) BMSC viability after shock wave stimulation with different pressure levels (1, 2, 3, and 4 bar) and impulse numbers (300, 600, 900, and 1200 impulses). Untreated BMSCs served as the control. (B) HUVEC viability following treatment with different concentrations (0, 20, 40, and 80 μg/mL) of sEVs derived from untreated BMSCs (sEVs) or shock wave-preconditioned BMSCs (SW-sEVs), as assessed by the CCK-8 assay. (C) Functional screening of sEVs derived from BMSCs exposed to different combinations of shock wave pressure (1–4 bar) and impulse number (300–1200 impulses) based on their effects on HUVEC viability. HUVECs were treated with 40 μg/mL sEVs in all groups. HUVEC viability was normalized to that of HUVECs treated with sEVs derived from untreated BMSCs (Control-sEVs), which was defined as 100%. Data are presented as mean ± SD (n = 3 independent experiments). *p < 0.05 and **p < 0.01 versus the corresponding 0 μg/mL group.

**Figure S2. Functional optimization of shock wave preconditioning parameters based on the pro-angiogenic activity of BMSC-derived sEVs.** (A) Representative images of HUVEC tube formation following treatment with 40 μg/mL sEVs derived from untreated BMSCs (Control-sEVs) or BMSCs preconditioned with shock waves at different pressures (1, 2, and 3 bar) and impulse numbers (300 and 600 impulses). Scale bar, 100 μm. (B) Quantitative analysis of total tube length. Data are presented as mean ± SD (n = 3 independent experiments). *p < 0.05 and **p < 0.01 versus the Control-sEV group.

**Figure S3. Quantitative analysis of tube formation assay.** The total tube length was quantified to evaluate the pro-angiogenic effects of sEVs and SW-sEVs. Compared with the control group, sEV treatment significantly increased total tube length, whereas SW-sEVs further enhanced tube formation relative to sEVs. Data are presented as mean ± SD (n = 3 independent experiments). **P < 0.01, ****P < 0.0001.

**Figure S4. Expression of angiogenesis-related proteins after treatment with sEVs and SW-sEVs.** (A) Representative western blot images showing the expression of VEGFR2 and VEGFA, with β-actin used as the loading control. (B, C) Quantitative analysis of VEGFR2 and VEGFA expression levels. Compared with the control group, sEVs increased the expression of angiogenesis-related proteins, while SW-sEVs further upregulated VEGFR2 and VEGFA expression. Data are presented as mean ± SD (n = 3 independent experiments). ns, not significant; **P < 0.01, ***P < 0.001.

**Figure S5. Morphological characterization of free and released SW-sEVs.** Representative transmission electron microscopy images showing the morphology of free SW-sEVs and released SW-sEVs. Both free and released SW-sEVs exhibited typical vesicle-like structures, indicating that the release process did not markedly alter the morphological characteristics of SW-sEVs. Scale bar, 50 nm. Data are representative of three independent experiments (n = 3).

**Figure S6. Quantitative analysis of PKH26-associated fluorescence corresponding to Figure 5J and K.** (A) Relative PKH26 fluorescence intensity in HUVECs at 24, 48, and 72 h following treatment with free SW-sEVs or GelMA-SW-sEVs. (B) Relative PKH26 fluorescence intensity in RAW264.7 macrophages following the corresponding treatments. Fluorescence intensity at 24 h was used as the reference for normalization. Data are presented as mean ± SD (n = 3 independent experiments).

**Figure S7. Histological evaluation of the in vivo biosafety of GelMA-based sEV delivery.** Representative hematoxylin and eosin (H&E) staining images of the heart, liver, spleen, lung, and kidney collected from rats in the Control, GelMA, GelMA-sEVs, and GelMA-SW-sEVs groups at the end of the treatment period (n = 6 rats per group). Scale bar, 50 μm.
